# 2-[(*E*)-4-(2-Bromo­phen­yl)but-3-en-2-yl­idene]malononitrile

**DOI:** 10.1107/S1600536810049536

**Published:** 2010-11-30

**Authors:** Tai-Ran Kang

**Affiliations:** a College of Chemistry and Chemical Engineering, China West Normal University, Nanchong 637002, People’s Republic of China

## Abstract

The title compound, C_13_H_19_BrN_2_, is planar structure except for the methyl H atoms, the maximum atomic deviation for the non-H atoms being 0.100 (1) Å. The bromo­phenyl and isopropanylidenemalononitrile units are located on opposite sides of the C=C bond, showing an *E* configuration.

## Related literature

For the use of malononitrile-containing compounds as building blocks in syntheses, see: Liu *et al.* (2002 ▶); Sepiol & Milart (1985 ▶); Zhang *et al.* (2003 ▶). For a related structure, see: Chen & Kang (2010 ▶).
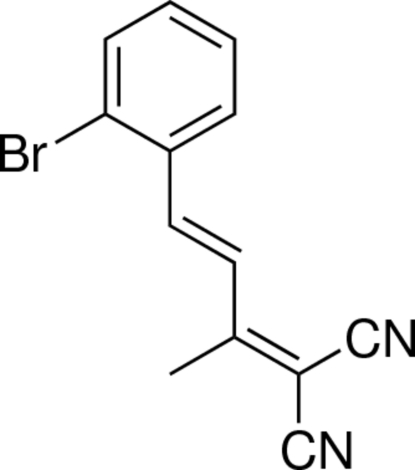


## Experimental

### 

#### Crystal data

C_13_H_9_BrN_2_
*M* *_r_* = 273.13
Triclinic, 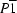

*a* = 7.0353 (7) Å
*b* = 7.0765 (5) Å
*c* = 13.3229 (8) Å
α = 82.192 (6)°
β = 76.628 (8)°
γ = 66.038 (9)°
*V* = 589.03 (8) Å^3^
*Z* = 2
Cu *K*α radiation
μ = 4.52 mm^−1^
*T* = 291 K
0.36 × 0.32 × 0.24 mm

#### Data collection

Oxford Diffraction Xcalibur Sapphire3 Gemini ultra diffractometer
Absorption correction: multi-scan (*CrysAlis PRO*; Oxford Diffraction, 2009 ▶) *T*_min_ = 0.293, *T*_max_ = 0.410
4500 measured reflections
2062 independent reflections
1923 reflections with *I* > 2σ(*I*)
*R*_int_ = 0.024

#### Refinement

*R*[*F* ^2^ > 2σ(*F* ^2^)] = 0.039
*wR*(*F* ^2^) = 0.112
*S* = 1.06
2062 reflections
146 parameters
H-atom parameters constrained
Δρ_max_ = 0.59 e Å^−3^
Δρ_min_ = −0.55 e Å^−3^

### 

Data collection: *CrysAlis CCD* (Oxford Diffraction, 2008 ▶); cell refinement: *CrysAlis RED* (Oxford Diffraction, 2008 ▶); data reduction: *CrysAlis RED*; program(s) used to solve structure: *SHELXS97* (Sheldrick, 2008 ▶); program(s) used to refine structure: *SHELXL97* (Sheldrick, 2008 ▶); molecular graphics: *ORTEP-3* (Farrugia, 1997 ▶); software used to prepare material for publication: *SHELXL97*.

## Supplementary Material

Crystal structure: contains datablocks global, I. DOI: 10.1107/S1600536810049536/xu5097sup1.cif

Structure factors: contains datablocks I. DOI: 10.1107/S1600536810049536/xu5097Isup2.hkl

Additional supplementary materials:  crystallographic information; 3D view; checkCIF report

## Figures and Tables

**Fig. 1.**
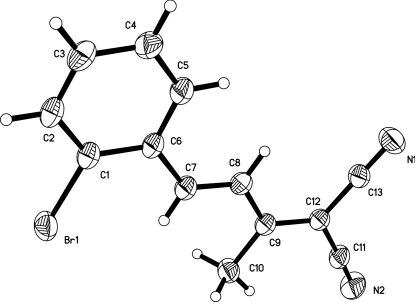
The molecular structure of (I) with 30% probability displacement ellipsoids (arbitrary spheres for H atoms).

## supplementary crystallographic information

### Comment

Malononitrile derivatives have broad application for the preparation of heterocyclic ring compounds. The chemistry of ylidene malononitrile have been studied extensively, From the ring closure reactions, the comounds containing newly formed five or six-membered rings, such as indans (Zhang *et al.*, 2003), naphthalenes (Liu *et al.*, 2002), benzenes (Sepiol *et al.*, 1985) were obtained. Some crystal structures involving ylidene malononitrile groups have been published, including a recent report from our labratory Chen, *et al.*, 2010). As a part of our interest in the synthsis of some complex ring systems, we investigated the title compound (I), which is a diene reagent in Diels-Alder reaction. We report herein the crystal structure of the title compound.

The molecular structure of (I) is shown in Fig. 1. Bond lengths and angles in (I) are normal. The molecule skeleton display an approximately planar structure except for the methyl group.

### Experimental

2-(Propan-2-ylidene)malononitrile (0.212 g, 2 mmol) and 2-bromobenzaldehyde (0.366 g, 2 mmol) were dissolved in 2-propanol (2 ml). To the solution was added piperidine (0.017 g, 0.2 mmol), the solution was stirred for 24 h at 343 K. Then the reaction was cooled to room temperature, and the solution was filtered to obtain a yellow solid. Recrystallization from hot ethanol afforded the pure compound. Single crystals of (I) suitable for X-ray analysis were obtained by slow evaporation ethyl acetate solution.

### Refinement

H atoms were placed in calculated positions with C—H = 0.93–0.96 Å, and refined using a riding model with *U*_iso_(H) = 1.5*U*_eq_(C) for methyl H atoms and *U*_iso_(H) = 1.2*U*_eq_(C) for the others.

### Crystal data

**Table:**

C_13_H_9_BrN_2_	*Z* = 2
*M**_r_* = 273.13	*F*(000) = 272
Triclinic, *P*1	*D*_x_ = 1.540 Mg m^−^^3^
Hall symbol: -P 1	Cu *K*α radiation, λ = 1.54184 Å
*a* = 7.0353 (7) Å	Cell parameters from 3664 reflections
*b* = 7.0765 (5) Å	θ = 6.8–71.9°
*c* = 13.3229 (8) Å	µ = 4.52 mm^−^^1^
α = 82.192 (6)°	*T* = 291 K
β = 76.628 (8)°	Block, yellow
γ = 66.038 (9)°	0.36 × 0.32 × 0.24 mm
*V* = 589.03 (8) Å^3^	

### Data collection

**Table:**

Oxford Diffraction Xcalibur Sapphire3 Gemini ultra diffractometer	2062 independent reflections
Radiation source: Enhance Ultra (Cu) X-ray Source	1923 reflections with *I* > 2σ(*I*)
mirror	*R*_int_ = 0.024
Detector resolution: 7.9575 pixels mm^-1^	θ_max_ = 67.0°, θ_min_ = 6.8°
ω scans	*h* = −8→6
Absorption correction: multi-scan (*CrysAlis PRO*; Oxford Diffraction, 2009)	*k* = −8→8
*T*_min_ = 0.293, *T*_max_ = 0.410	*l* = −15→15
4500 measured reflections	

### Refinement

**Table:**

Refinement on *F*^2^	Primary atom site location: structure-invariant direct methods
Least-squares matrix: full	Secondary atom site location: difference Fourier map
*R*[*F*^2^ > 2σ(*F*^2^)] = 0.039	Hydrogen site location: inferred from neighbouring sites
*wR*(*F*^2^) = 0.112	H-atom parameters constrained
*S* = 1.06	*w* = 1/[σ^2^(*F*_o_^2^) + (0.0759*P*)^2^ + 0.0933*P*] where *P* = (*F*_o_^2^ + 2*F*_c_^2^)/3
2062 reflections	(Δ/σ)_max_ < 0.001
146 parameters	Δρ_max_ = 0.59 e Å^−^^3^
0 restraints	Δρ_min_ = −0.55 e Å^−^^3^

### Special details

**Table:**

Geometry. All e.s.d.'s (except the e.s.d. in the dihedral angle between two l.s. planes) are estimated using the full covariance matrix. The cell e.s.d.'s are taken into account individually in the estimation of e.s.d.'s in distances, angles and torsion angles; correlations between e.s.d.'s in cell parameters are only used when they are defined by crystal symmetry. An approximate (isotropic) treatment of cell e.s.d.'s is used for estimating e.s.d.'s involving l.s. planes.
Refinement. Refinement of *F*^2^ against ALL reflections. The weighted *R*-factor *wR* and goodness of fit *S* are based on *F*^2^, conventional *R*-factors *R* are based on *F*, with *F* set to zero for negative *F*^2^. The threshold expression of *F*^2^ > σ(*F*^2^) is used only for calculating *R*-factors(gt) *etc*. and is not relevant to the choice of reflections for refinement. *R*-factors based on *F*^2^ are statistically about twice as large as those based on *F*, and *R*- factors based on ALL data will be even larger.

### Fractional atomic coordinates and isotropic or equivalent isotropic displacement parameters (Å^2^)

**Table:**

	*x*	*y*	*z*	*U*_iso_*/*U*_eq_	
Br1	0.67339 (5)	0.15902 (4)	0.54256 (2)	0.06643 (19)	
C8	0.8775 (4)	−0.3609 (4)	0.7789 (2)	0.0493 (6)	
H8	0.8557	−0.3515	0.8499	0.059*	
N2	1.2571 (5)	−1.0939 (4)	0.7188 (2)	0.0735 (8)	
C11	1.0519 (4)	−0.7373 (4)	0.7984 (2)	0.0455 (5)	
C4	0.5298 (5)	0.2647 (5)	0.8955 (2)	0.0598 (7)	
H4	0.4994	0.2887	0.9655	0.072*	
C13	1.0087 (4)	−0.7342 (4)	0.9092 (2)	0.0527 (6)	
C5	0.6402 (5)	0.0655 (4)	0.8620 (2)	0.0563 (6)	
H5	0.6835	−0.0433	0.9103	0.068*	
C9	0.9905 (4)	−0.5636 (4)	0.7362 (2)	0.0461 (5)	
C7	0.8037 (4)	−0.1879 (4)	0.7217 (2)	0.0516 (6)	
H7	0.8277	−0.2024	0.6510	0.062*	
C2	0.5107 (4)	0.3937 (4)	0.7208 (3)	0.0563 (7)	
H2	0.4684	0.5041	0.6732	0.068*	
C10	1.0389 (5)	−0.5797 (5)	0.6211 (2)	0.0584 (7)	
H10A	1.1160	−0.7222	0.6044	0.088*	
H10B	1.1228	−0.5013	0.5895	0.088*	
H10C	0.9087	−0.5260	0.5958	0.088*	
C6	0.6889 (4)	0.0226 (4)	0.7573 (2)	0.0478 (6)	
N1	0.9737 (5)	−0.7329 (5)	0.9974 (2)	0.0749 (8)	
C3	0.4644 (4)	0.4289 (4)	0.8242 (3)	0.0604 (7)	
H3	0.3891	0.5629	0.8466	0.072*	
C12	1.1667 (4)	−0.9376 (4)	0.7555 (2)	0.0532 (6)	
C1	0.6206 (4)	0.1936 (4)	0.6873 (2)	0.0496 (6)	

### Atomic displacement parameters (Å^2^)

**Table:**

	*U*^11^	*U*^22^	*U*^33^	*U*^12^	*U*^13^	*U*^23^
Br1	0.0880 (3)	0.0444 (2)	0.0549 (3)	−0.01383 (17)	−0.01666 (17)	0.00310 (15)
C8	0.0616 (14)	0.0331 (13)	0.0509 (14)	−0.0141 (11)	−0.0134 (11)	−0.0042 (10)
N2	0.0868 (17)	0.0364 (14)	0.0813 (19)	−0.0086 (12)	−0.0094 (14)	−0.0120 (13)
C11	0.0542 (12)	0.0334 (12)	0.0467 (13)	−0.0140 (10)	−0.0109 (10)	−0.0024 (10)
C4	0.0712 (16)	0.0478 (15)	0.0555 (16)	−0.0208 (13)	−0.0032 (13)	−0.0105 (12)
C13	0.0673 (15)	0.0354 (13)	0.0519 (17)	−0.0138 (11)	−0.0196 (12)	0.0028 (11)
C5	0.0706 (16)	0.0384 (14)	0.0559 (16)	−0.0179 (12)	−0.0128 (12)	0.0009 (12)
C9	0.0549 (12)	0.0336 (12)	0.0481 (14)	−0.0145 (10)	−0.0117 (10)	−0.0024 (10)
C7	0.0654 (14)	0.0336 (13)	0.0522 (15)	−0.0145 (11)	−0.0141 (11)	−0.0015 (11)
C2	0.0583 (14)	0.0329 (13)	0.0699 (19)	−0.0105 (11)	−0.0140 (13)	0.0022 (12)
C10	0.0780 (17)	0.0425 (15)	0.0469 (15)	−0.0169 (13)	−0.0093 (13)	−0.0031 (11)
C6	0.0539 (13)	0.0318 (12)	0.0551 (15)	−0.0140 (10)	−0.0119 (11)	0.0003 (10)
N1	0.105 (2)	0.0617 (17)	0.0521 (17)	−0.0232 (15)	−0.0236 (14)	0.0012 (12)
C3	0.0602 (15)	0.0371 (14)	0.076 (2)	−0.0120 (11)	−0.0060 (13)	−0.0109 (13)
C12	0.0629 (15)	0.0354 (14)	0.0566 (16)	−0.0150 (12)	−0.0122 (12)	0.0011 (12)
C1	0.0508 (12)	0.0359 (13)	0.0575 (15)	−0.0125 (10)	−0.0116 (11)	0.0009 (11)

### Geometric parameters (Å, °)

**Table:**

Br1—C1	1.908 (3)	C5—H5	0.9300
C8—C7	1.327 (4)	C9—C10	1.502 (4)
C8—C9	1.449 (4)	C7—C6	1.461 (4)
C8—H8	0.9300	C7—H7	0.9300
N2—C12	1.140 (4)	C2—C3	1.373 (5)
C11—C9	1.358 (4)	C2—C1	1.387 (4)
C11—C12	1.437 (4)	C2—H2	0.9300
C11—C13	1.438 (4)	C10—H10A	0.9600
C4—C5	1.383 (4)	C10—H10B	0.9600
C4—C3	1.390 (5)	C10—H10C	0.9600
C4—H4	0.9300	C6—C1	1.412 (4)
C13—N1	1.144 (4)	C3—H3	0.9300
C5—C6	1.401 (4)		
			
C7—C8—C9	123.3 (3)	C3—C2—C1	120.0 (3)
C7—C8—H8	118.4	C3—C2—H2	120.0
C9—C8—H8	118.4	C1—C2—H2	120.0
C9—C11—C12	120.8 (2)	C9—C10—H10A	109.5
C9—C11—C13	123.1 (2)	C9—C10—H10B	109.5
C12—C11—C13	116.1 (2)	H10A—C10—H10B	109.5
C5—C4—C3	119.7 (3)	C9—C10—H10C	109.5
C5—C4—H4	120.2	H10A—C10—H10C	109.5
C3—C4—H4	120.2	H10B—C10—H10C	109.5
N1—C13—C11	179.5 (3)	C5—C6—C1	116.6 (2)
C4—C5—C6	121.9 (3)	C5—C6—C7	122.1 (2)
C4—C5—H5	119.0	C1—C6—C7	121.3 (3)
C6—C5—H5	119.0	C2—C3—C4	120.2 (3)
C11—C9—C8	121.1 (2)	C2—C3—H3	119.9
C11—C9—C10	120.0 (2)	C4—C3—H3	119.9
C8—C9—C10	119.0 (2)	N2—C12—C11	178.1 (3)
C8—C7—C6	127.4 (3)	C2—C1—C6	121.5 (3)
C8—C7—H7	116.3	C2—C1—Br1	117.1 (2)
C6—C7—H7	116.3	C6—C1—Br1	121.4 (2)
			
C9—C11—C13—N1	131 (44)	C8—C7—C6—C5	−0.8 (5)
C12—C11—C13—N1	−49 (45)	C8—C7—C6—C1	179.5 (3)
C3—C4—C5—C6	−0.1 (5)	C1—C2—C3—C4	0.9 (4)
C12—C11—C9—C8	−179.0 (2)	C5—C4—C3—C2	−0.5 (5)
C13—C11—C9—C8	0.7 (4)	C9—C11—C12—N2	−6(10)
C12—C11—C9—C10	1.1 (4)	C13—C11—C12—N2	175 (10)
C13—C11—C9—C10	−179.1 (3)	C3—C2—C1—C6	−0.6 (4)
C7—C8—C9—C11	−176.0 (3)	C3—C2—C1—Br1	178.9 (2)
C7—C8—C9—C10	3.8 (4)	C5—C6—C1—C2	0.0 (4)
C9—C8—C7—C6	−179.9 (3)	C7—C6—C1—C2	179.7 (3)
C4—C5—C6—C1	0.4 (4)	C5—C6—C1—Br1	−179.5 (2)
C4—C5—C6—C7	−179.4 (3)	C7—C6—C1—Br1	0.2 (3)
